# Post-Pulmonary Embolism Syndrome: New Phenotypes Come into Focus

**DOI:** 10.3390/jcm15020635

**Published:** 2026-01-13

**Authors:** Bilal H. Lashari, Stephen Dachert, Belinda N. Rivera-Lebron, Brandon Hooks, Parth Rali

**Affiliations:** 1 Department of Thoracic Medicine and Surgery, Lewis Katz School of Medicine, Temple University, Philadelphia, PA 19140, USA; 2 Division of Pulmonary and Critical Care Medicine, Department of Medicine, University of Pittsburgh, Pittsburgh, PA 15260, USA; 3 Division of Pulmonary Disease and Critical Care Medicine, Department of Medicine, University of Michigan Health West, Wyoming, MI 49519, USA

**Keywords:** dyspnea, pulmonary embolism, post pulmonary embolism syndrome, quality of life, chronic thromboembolic pulmonary disease, chronic thromboembolic pulmonary hypertension

## Abstract

The acute phase of pulmonary embolism (PE) may be a severe and potentially life-threatening condition. Moreover, long-term consequences following the acute phase can significantly impact a patient’s daily life. A systematic approach to PE follow-up can identify potential complications following acute PE. Post-PE syndrome (PPES) is a common occurrence among survivors experiencing persistent dyspnea and impaired functional status. While the exact definition is evolving, it encompasses a spectrum of disease phenotypes that may occur following an acute PE, which ranges from dyspnea, functional limitation, or cardiac impairment to chronic thromboembolic disease and chronic thromboembolic pulmonary hypertension. This review will describe the different PPES phenotypes, including their physiological basis, diagnosis and workup, and management following acute PE.

## 1. Introduction

### 1.1. Definition

Venous thromboembolism (VTE) affects around 900,000 people yearly in the United States alone, and it is the third leading cause of cardiovascular mortality in the world [1,2,3,4]. In recent years, there has been growing focus on management of acute PE, with advances in diagnostic techniques, sophisticated risk stratification, and increased therapeutic alternatives [5,6]. However, as clinicians become more versed in acute inpatient PE care, it is important to consider the post-acute phase, its long-term outcomes, and its quality of life and morbidity implications.

Approximately half of patients that have had an acute PE will experience some degree of exercise intolerance and dyspnea post PE [6]. Up to 30% of patients have persistent clots, 10–30% have cardiopulmonary limitation, and 0.5–4% will develop chronic thromboembolic pulmonary hypertension (CTEPH) [7,8]. While its definition is still evolving, the term post-PE syndrome (PPES) has been used recently to describe a range of subjective symptoms of persistence of dyspnea, exercise limitation, and impaired quality of life (QoL), as well as objective cardiopulmonary limitations following an acute PE [9]. While pathophysiology and management are not well understood, close follow-up is crucial given its implications in morbidity and mortality. PPES encompasses post-PE dyspnea or cardiac impairment, chronic thromboembolic pulmonary disease (CTEPD), and CTEPH. Herein, we will discuss the evolving phenotypes of PPES, including diagnosis, management, and prognostic implications.

### 1.2. Pathobiology Post PE

After an acute PE, the principal components of an acute thrombus, namely erythrocytes and fibrin, are naturally degraded. Fibrin degradation into d-dimers starts soon after the formation of a thrombus and is evident in 80% of patients within a week, up to 40% within 1 month, and only 13% at 3 months [10]. Erythrocytes usually expire within 2–4 months and are degraded by circulating macrophages. Impaired fibrinolysis, altered fibrinogen structure and function, increased local or systemic inflammation, and remodeling of the embolic material by neovascularization have been implicated in failure of clot resolution. Inflammation and hypoxia-induced signaling appear to be central to the persistence of thrombotic disease and failure of clot resolution, as evidenced by the elevation in macrophages and T-cell-mediated inflammation, as well as elevated circulating inflammatory mediators such as CRP, TNF-a, CXCL-10, IL-2, IL-4, IL-8, and IL-10. 

### 1.3. Phenotypes of Post-Pulmonary Embolism Syndrome

Table 1 provides the various clinical phenotypes of PPES. Post-PE dyspnea or functional impairment is defined as the persistence of dyspnea, exercise intolerance, or decreased functional status after an acute PE, in the absence of an alternate explanation. It is postulated that decreased physical activity after PE and deconditioning contribute to the persistence of exercise intolerance in most patients [11]. Pain, anxiety, fear of recurrence, and comorbidities, such as obesity and other cardiopulmonary conditions, may also contribute and lead to the avoidance of exercise and reduced quality of life. When compared to patients without a history of venous thromboembolism (VTE), patients with an acute episode of PE are more likely to report NYHA class II (OR 3.6 95%CI 1.4–9.7) and class III or IV (OR 6.5 95%CI 1.7–24) dyspnea, when controlled for gender and age [12,13]. Across multiple studies, survivors of an acute PE report a lower QoL secondary to dyspnea [14].

Persistent right ventricular (RV) hypokinesis, dysfunction, or dilation without evidence of pulmonary hypertension (PH), in the absence of an alternate explanation, after 3 months following acute PE is termed post-PE cardiac impairment [15]. Up to 50% of all patients with acute PE may present with cardiac impairment [16], and up to a fourth may have persistent impairment after three months [17]. Despite timely and effective anticoagulation in the acute setting, a combination of initial ischemic insult, inflammation, and structural remodeling has been implicated in this persistent dysfunction [8,18,19,20].

CTEPD refers to persistent thrombosis causing pulmonary vascular obstruction without PH at rest measured on a right heart catheterization (RHC). Complete radiographic resolution of thrombus on a computed tomography angiogram (CTA) was reported in 84% of patients at 6 months post diagnosis. Another study reported persistent perfusion defects on a Ventilation/Perfusion (VQ) scan in 29% of patients after 1 year following adequate anticoagulation. Clot persistence leads to functional dead space, disturbances in gas exchange, and ineffective ventilation. CTEPD symptoms may range from mild dyspnea to severe cardiopulmonary limitations with evidence of PH with exercise. Older age (>65 years), unprovoked PE, a diagnosis of chronic obstructive pulmonary disease (COPD), chronic respiratory failure, and VTE are independent risk factors for its development [10,11].

The most severe complication following acute PE is CTEPH. It is defined as persistent thrombosis causing resting pulmonary arterial hypertension (mean pulmonary artery pressure (mPAP) above 20 mmHg, pulmonary artery wedge pressure (PCWP) of less than 15 mmHg, and pulmonary vascular resistance (PVR) equal to or greater than 2, on an RHC). Non-resolving organizing thrombus and microvascular changes in the circulation lead to increased PVR and development of PH. The development of CTEPH ranges from 0.4 to 10% in patients that survived an episode of acute PE.

The FOCUS study, a large prospective multicenter observational study conducted across multiple tertiary care centers in Germany, tracked 1017 patients with acute PE over a two-year follow-up period. The estimated incidence of CTEPH was 2.3%. More notably, the study identified that 116 patients (13.2%) experienced post-PE impairment, characterized by either a worsening or a persistently high severity in echocardiographic findings alongside at least one clinical, functional, or laboratory parameter. These assessments were conducted during follow-up visits at 3, 12, or 24 months, providing a comprehensive view of the patients’ health trajectory post PE [13].

## 2. Diagnosis

Outpatient follow-up after acute PE with a thorough interview is indispensable to identify PPES and guide the clinician in determining follow-up testing. The timing of follow-up visits depends on the patient’s severity at presentation and hospital resources. The Pulmonary Embolism Response Team (PERT) Consortium consensus guidelines on the treatment and follow-up of acute PE recommends a visit between 2 weeks and 3 months after acute PE, while the European Society of Cardiology (ESC) guidelines suggest a visit between 3 and 6 months [5,21].

The initial visit should include symptom evaluation, review of anticoagulation (dosing, possible drug interactions, expected duration), establishment of the need for follow-up testing along with the timeline, and review of indications for additional testing, such as hypercoagulable disorders and age-appropriate cancer screening. Patients should be evaluated for persistent dyspnea, decreased exercise tolerance, chest pain, dizziness, edema, lightheadedness, and/or syncope, and if present, follow-up testing should be pursued. Most patients do not repeat testing before 3 months of effective anticoagulation.

### 2.1. Symptom Evaluation

Symptom screening, focusing on dyspnea and exercise tolerance, is performed using directed questions, regardless of the PE severity during original presentation. Validated tools such as the modified Medical Research Council (mMRC) score [22] help provide a comparison to the pre-PE baseline. The SF-36 questionnaire consists of 36 questions that assess a patient’s perceptions of their physical and mental health over the preceding four weeks [23]. The PE Quality of Life Questionnaire (PEmb-QOL) is a validated forty-question tool that covers six areas, including the frequency and severity of symptoms, and how they affect the patient’s emotions, daily activities, work, and social life [24], that may be useful in the identification of patients requiring further evaluation [25]. A recently developed scale, the post-venous thromboembolism functional status (PVFS) scale [26], includes the level of care a person may require, ability to complete activities of daily living, participation in social roles, and any residual symptoms.

Each tool has its unique characteristics and limitations when used in the identification of PPES. The mMRC score is highly sensitive in the detection of dyspnea severity in symptomatic patients; however, it is limited in identifying patients with objective exercise limitation in the absence of symptoms. The SF-36 is good at capturing generic physical and mental health deficits but is not specific to PPES. PEmb-QoL, however, is PE-specific and is a reliable instrument that can be cumbersome to administer given its 40 scoring items. In contrast, the PVFS is practical and brief but has limited direct validation in PPES.

Routine use of most functional scores may be challenging to implement in clinical practice, but overall, these scores highlight the importance of symptom-based screening for post-PE phenotypes.

### 2.2. Psychological Sequelae After PE

The psychological impact of PE is a key component of post-PE syndrome and represents an important but underrecognized domain of survivorship. Pulmonary embolism (PE) is increasingly recognized not only as a life-threatening acute event but also as a condition with substantial long-term sequelae. Beyond the well-documented physical consequences such as post-PE dyspnea, exercise limitation, and CTEPH, survivors frequently experience profound psychological effects. These sequelae include anxiety, depression, and post-traumatic stress disorder (PTSD)-like symptoms, which may persist long after resolution of the acute thrombotic episode. Collectively, these manifestations are encompassed within the framework of post-PE syndrome.

Although ongoing research continues to explore the psychological impact of venous thromboembolism (VTE), significant gaps remain in our understanding of emotional and mental well-being in this population. Multiple studies have demonstrated that patients with PE have higher rates of depression and anxiety compared with the general population [27]. Survivors often report poor mental health as measured by health-related quality of life questionnaires [28], with mood disturbances compounding the physical limitations imposed by the thromboembolic event [29].

PE is frequently described by patients as a “life-changing diagnosis”. Survivors may articulate a sense of lost identity, intrusive thoughts, and the need to adjust to new functional restrictions. Emotional responses often include insecurity about recognizing or managing future symptoms, perceived loss of invincibility, and shifts in self-perception [30]. Many individuals describe living in a state of hypervigilance, characterized by persistent worry or fear of recurrence. The symptoms of hypervigilance and fear of recurrence often lead patients to question whether prescribed medications are working. In addition, the development of symptoms similar to those of initial VTE (dyspnea, leg pain, chest discomfort), for example, may lead to unnecessary imaging and use of resources such as repeated emergency room evaluations.

One specific psychological response increasingly described in the literature is post-thrombotic panic syndrome, defined by hypervigilance to bodily sensations and cyclically triggered emotional distress. Patients report fear of recurrence, flashbacks, and intrusive thoughts related to their initial PE event. These symptoms, in severe forms, may meet diagnostic criteria for PTSD [31].

PTSD following PE is not uncommon and may present with avoidance behaviors, nightmares, hyperarousal, and emotional numbing. If unrecognized, these symptoms can severely impair recovery, social reintegration, and overall quality of life. Early recognition and systematic assessment are therefore critical.

During follow-up visits, clinicians should explicitly inquire about emotional health, including symptoms of anxiety, depression, and PTSD-like features. These symptoms often emerge within the first few months after diagnosis but can persist for years if untreated. Early identification allows for timely referral to supportive interventions. The Pulmonary Embolism Quality of Life (PEmb-QoL) Questionnaire captures both physical and emotional sequelae and can be utilized as a structured screening to help identify patients who may benefit from referral to mental health professionals [32].

In the ELOPE study, a study of 100 consecutive patients that were followed up with post PE, patients had an overall improvement in their mean SF-36 physical and mental components, and PEmb-QoL, over the course of the year following an acute PE.

In addition to dyspnea and functional limitations, psychological complications following acute PE diagnosis are also common. In a recent large German cohort, one in five patients diagnosed with acute PE were also found to have depression or anxiety. Risk factors for its development were older age, higher simplified PE score index (sPESI), higher education level, lower oxygen saturation, and longer hospitalization [33,34], as well as patients’ lack of knowledge on post-PE care [35].

## 3. Exercise Capacity Evaluation

### 3.1. Six-Minute Walk Test

The 6 min walk test (6MWT) is a simple measure of exercise capacity utilized in clinical practice and research that has been validated to predict outcomes in pulmonary vascular disease [36]. In a study of 205 patients with intermediate-risk PE, individuals underwent a 6MWT and echocardiography six months after the initial event. Findings revealed that 41% had cardiopulmonary abnormalities at follow-up: 17% showed abnormal RV function, 17% had functional limitations in the 6MWT, and 8% had both. Patients with these abnormalities experienced a significant drop in oxygen saturation (SaO_2_%) during the 6MWT [8]. Similarly, patients without dyspnea following an acute PE performed better on a 6MWT (488 m vs. 413 m, *p* < 0.005) and had better HRQoL results when compared to patients that complained of dyspnea [37].

### 3.2. Cardiopulmonary Exercise Testing

A cardiopulmonary exercise test (CPET) is a noninvasive method to assess functional capacity and exercise limitation. During a CPET, gas exchange abnormalities are related to alveolar hypoperfusion relative to ventilation [12,33]. Post-PE functional impairment may be characterized by a normal or near-normal physiologic dead space (VD/VT) and a reduced oxygen uptake (VO_2_) at an anaerobic threshold, indicating deconditioning as a major contributor to post-PE functional impairment [33,38,39]. A reduced VO_2_ max, seen typically in left ventricular dysfunction, may not be predictive of RV dysfunction or defects in lung perfusion, but may be indicative of inadequate cardiac contractility to surmount increased pulmonary pressures for chronic vascular obstruction. With exercise, patients with CTEPD may have reduced peak oxygen uptake (VO_2_), elevated dead space (VD/VT), impaired perfusion (lower end-tidal carbon dioxide (PETCO_2_)), a reduced anaerobic threshold (AT), inefficient ventilation (increased ⩒e/⩒CO_2_), and limited ability to increase stroke volume [16].

In the prospective ELOPE cohort of 100 post-PE patients, 47% had exercise limitation measured by peak VO_2_ < 80% at 1 year. Additionally, these patients had worse 6MWT and QOL metrics compared to those with a predicted VO_2_ > 80% [27].

In a subgroup of the large prospective cohort FOCUS, patients with acute symptomatic PE, 396 patients underwent CPET at 3 months and 268 patients at 12 months post acute PE. Fifty percent had at least one abnormal parameter indicating cardiopulmonary limitation. Of patients undergoing CPET at both time points, 16.7% had resolving cardiopulmonary limitation and 24.8% developed a new limitation. Of those experiencing post-PE impairment, 79.1% and 78.6% had cardiopulmonary limitation at three and six months, respectively. Definitions of CPET abnormalities in PPES can be found in Table 2 [40].

### 3.3. Cardiac Function Evaluation: Echocardiography

Multiple studies have demonstrated structural heart changes, specifically RV dysfunction, following acute PE in previously healthy patients. In 109 previously healthy patients, 27 patients (25%) had abnormal RV function six months after acute PE [8]. Additionally, a large cohort of 845 patients showed residual RV dysfunction in 27% of symptomatic PE survivors six months following acute PE [41].

Severity of PE at diagnosis may also affect RV function at follow-up. In a cohort of 20 patients undergoing CPET after an intermediate- or high-risk PE, 65% continued to show RV dilation or dysfunction one month after acute PE, persisting six months later [38]. The REACH study performed in 2020 demonstrated that in 508 patients, intermediate-risk PE was associated with higher likelihood of persistent RV dysfunction (44%) three months post-PE compared to a standard-risk group (18%) despite a low rate of CTEPH (0.6%) in this cohort [40]. Furthermore, in a prospective cohort of 144 PE survivors, 27% of patients had higher RV systolic pressure (RSVP) six months following the PE diagnosis [18].

Echocardiograms are pivotal for identifying or excluding alternate causes of symptoms. In a study of 555 patients treated for acute PE six months prior, 42 (7.5%) had reduced EF (<50%), 50 (9%) presented with valvular heart disease, and 265 (48%) showed LV diastolic dysfunction [41,42].

Findings of persistent RV dysfunction in the months to years following acute PE are independently associated with symptoms of dyspnea [20] and correlate with worse functional status [43]. A post hoc analysis of the PEITHO trial in 2019 showed that 10% of survivors of intermediate-risk PE experienced echocardiographic evidence of PH and NYHA II-IV symptoms [43].

## 4. Residual Thrombosis Evaluation: CT Pulmonary Angiography and Ventilation/Perfusion Scan

### 4.1. CT Pulmonary Angiography (CTA)

Incomplete clot resolution of acute PE may be seen on serial pulmonary imaging following acute PE. Residual clot burden following acute PE has also been studied using serial CTA following 6 months of uninterrupted anticoagulation. In a prospective cohort of 157 patients, complete PE resolution was seen in 84% of the patients. Ten percent of patients experienced recurrent VTE, but interestingly, the presence of residual pulmonary vascular obstruction (RPVO) was not associated with recurrent VTE. Prior VTE showed an increased risk of incomplete resolution [6].

### 4.2. Ventilation/Perfusion Scan (VQ Scan)

In one study, 647 consecutive patients following the first episode of acute PE on anticoagulation were assessed for RPVO with a VQ scan after 6 months. It showed persistent perfusion defects in 50% of patients. Older age, unprovoked clinical presentation, and worse clinical severity during the acute PE episode were significantly associated with persistence of pulmonary obstruction. Additionally, in this cohort, after 3 years of follow-up, recurrent VTE and/or CTEPH developed in 10% of patients with RPVO and in 4% of patients without RPVO. Here, the presence of RPVO was an independent predictor of recurrent VTE and/or CTEPH [44].

Another prospective observational study of consecutive acute PE patients that had received anticoagulation for at least 3 months showed perfusion defects on a VQ scan in 29% of patients after a median follow-up of 12 months. In the study, 30% of patients were receiving anticoagulation at one year following acute PE. Independent risk factors for RPVO defects included older age, longer time between symptom onset and PE diagnosis, higher levels of initial vascular obstruction, and previous VTE [45].

Single-photon emission computed tomography (SPECT) VQ has a similar sensitivity to planar VQ scan; however, it has a higher specificity in the diagnosis of PE [46]. Therefore, it is a preferable modality by comparison as it is associated with fewer non-diagnostic studies [47]. VQ scans have been shown to be more sensitive than CTA in detecting chronic thromboembolic disease [48,49].

### 4.3. Summary of Diagnosis

Perfusion defects lead to increased alveolar dead space and VQ mismatching. The combination of these abnormalities in conjunction with persistent RV dysfunction results in CTEPD and/or CTEPH.

Table 3 provides a summary of findings of diagnostic tests in PPES phenotypes. As shown, symptoms in the months to years following acute PE diagnosis are common [50]. There is no single test to diagnose the presence of PPES phenotypes. One could argue for obtaining tests to evaluate exercise capacity, cardiac function, and residual thrombosis in all patients, given the shown increased risk of CTEPH, and its increased mortality. Other clinicians may order selective testing based on symptom severity, clot burden, and RV dysfunction on initial diagnosis. There have been several algorithms proposed for testing following acute PE diagnosis [5,21].

Three notable strategies for post-PE follow-up and PPES screening are (1) the SEARCH algorithm [51], (2) the FOCUS trial [13], and (3) the ESC/ERS algorithm for early CTEPH diagnosis [52].

The SEARCH algorithm (symptom screen, exercise function (CPET), arterial perfusion (VQ scan), resting heart function (echocardiogram), confirmatory imaging (CTA), and hemodynamics) is a stepwise algorithm that sorts patients by a hierarchical series of dichotomous tests into discreet categories of PPES [51]. A study for the validation of the SEARCH algorithm is ongoing [53].

An advantage of the SEARCH algorithm is that it is simple to use, organized in a stepwise fashion, and therefore may avoid unnecessary testing. On the other hand, a challenge may be the widespread availability of CPET testing and interpretation. Additionally, some clinicians may be hesitant to order CPET testing prior to cardiac or imaging evaluation.

Strategies for detection of PPES are discussed in the FOCUS trial. Here, authors did not establish a stepwise algorithm for post-PE follow-up, instead utilizing a composite of testing to better define post-PE impairment. At regular intervals (3, 12, and 24 months) following acute PE diagnosis, the authors suggest performing symptom, functional, laboratory, and echocardiographic testing [13]. Post-PE impairment was defined by deterioration (compared to findings at discharge, or to the previous follow-up visit) in at least one category, persistence of the abnormal finding on an echocardiogram (RV basal diameter, right atrium (RA) end-systolic area, tricuspid annulus plane systolic excursion (TAPSE), LV eccentricity index, estimated RA pressure, systolic tricuspid regurgitation (TR) jet velocity, or pericardial effusion), and/or clinical (progression of existing symptoms, clinical evidence of RV failure, syncope), functional (WHO functional class, 6MWT, CPET), or laboratory parameters (BNP, NT-proBNP) of RV failure. The median follow-up was 2 years. While the estimated cumulative incidence of CTEPH was low (2.3%), the incidence of post-PE impairment was 16% (95% CI 12.8–20.8%). Additionally, post-PE impairment helped identify 15 of the 16 patients diagnosed with CTEPH [13].

A subgroup of patients from the FOCUS cohort (396 of 1017) underwent CPET at 3 and 12 months. The prevalence of cardiopulmonary limitation (defined as ventilatory inefficiency or insufficient cardiocirculatory reserve) was 50% and 44% at 3 and 12 months, respectively, and that of deconditioning (defined as a peak O_2_ uptake <80%) was 12% and 15% at 3 and 12 months, respectively. The authors concluded that CPET can be considered for selected patients with persisting symptoms after PE [40].

The advantage of using the FOCUS trial for PPES screening is that it engages routine symptoms and testing that are already utilized for most post-PE follow-up. The benefit of the prespecified, standardized follow-up schedule and defined composite criteria to screen for PPES is a higher yield and earlier identification of patients than traditional follow-up methods, leading to flagging of patients at higher risk of rehospitalization, mortality, and worse quality of life, enabling risk stratification for follow-up intensity or interventions.

Here, follow-up testing emphasizes subjective and objective parameters of functional status, rather than the presence of residual thrombosis on imaging. Notably, VQ scans and CTA were not recommended. Additionally, this strategy encourages a longer follow-up period of 2 years, with repeated reassessments.

ESC/ERS guidelines recommend an algorithm for the diagnosis of CTEPH, although not specifically for PPES. Step one of this algorithm includes assessment of symptoms, a physical exam, BNP/NT-proBNP, and resting ECG testing. Step two recommends obtaining echocardiograms and VQ scans as the initial diagnostic tests [50]. A negative VQ scan has a high negative predictive value and can safely exclude CTEPH [49]. Echocardiographic probability of PH is based on tricuspid valve regurgitation (TVR). Patients with low probability of PH without risk factors should be investigated for an alternative etiology for dyspnea. Patients with risk factors and/or intermediate probability for PH should have repeated echocardiography follow-up, and patients with risk factors for PH and high probability of PH should undergo further intervention/RHC if indicated. According to the ESC/ERS algorithm, CPET testing should be performed in cases where echocardiographic testing is without abnormalities [52]. The advantage of the ESC/ERS algorithm strategy is that it recommends both imaging (with VQ scan) and RV function assessment (with echocardiogram) simultaneously. A challenge may be the widespread availability of VQ scans across centers.

**Figure 1:**
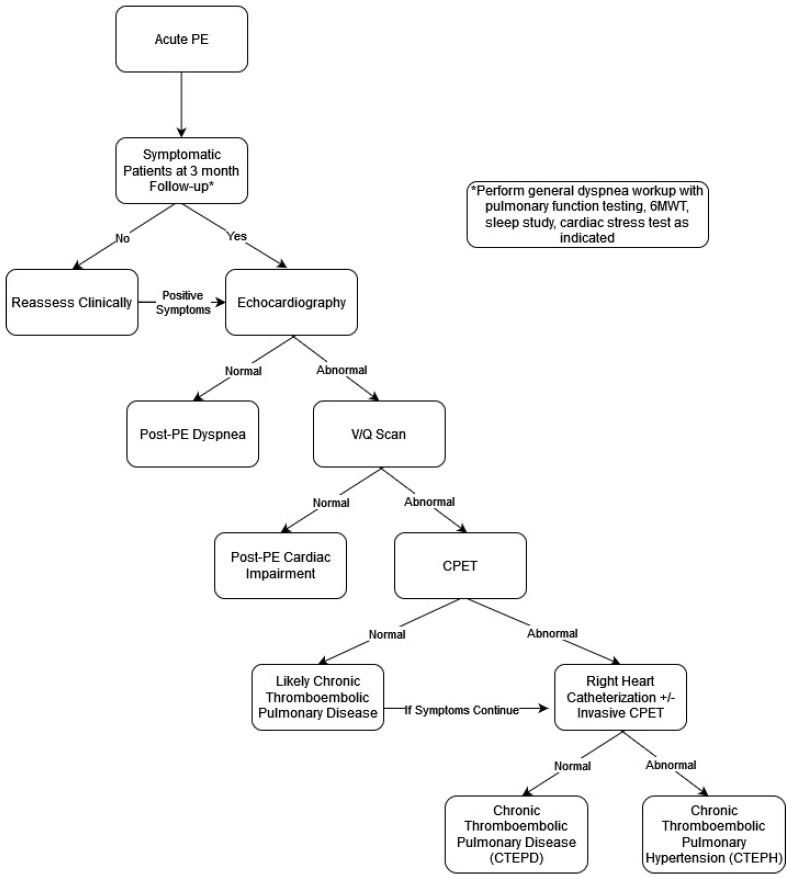
Schema for diagnosis of PPES Phenotypes.

Our proposed schema (Figure 1) for diagnosis of PPES incorporates elements from the mentioned diagnostic models. Patients diagnosed with acute PE should have a follow-up months post diagnosis. Initial screening for PPES should be focused on symptom assessment (with or without a validated questionnaire). Patients without symptoms should be counseled on seeking further care if they are to become symptomatic, especially those with abnormal RV or a large clot burden in the acute phase. Patients who are symptomatic should undergo echocardiography given its widespread availability and clinician comfort with ordering and interpretation. Additionally, at this time, symptomatic patients should also undergo generalized dyspnea workup with pulmonary function testing, a 6MWT, a sleep study, and/or a cardiac stress test, as indicated by their history and physical exam. Patients with normal echocardiography fit into the “Post-PE Dyspnea” phenotype. Patients with abnormal echocardiography should proceed with a VQ scan. Patients with a normal VQ test fit into the “Post-PE Cardiac Impairment” phenotype. Patients with an abnormal VQ scan should then undergo CPET if available. Patients with abnormal CPET should undergo RHC (resting or exercise/invasive, depending on availability and patients’ pre-test probability of having PH). Patients with normal resting RHC fit into the “CTEPD” phenotype. Patients with normal CPET may still fit into the CTEPD phenotype, but strong consideration should be given to exercise/invasive RHC, especially if symptoms continue. Patients with abnormal RHC fit into the “CTEPH” phenotype (Figure 1).

## 5. Management of Post-PE Syndrome

There is no clear treatment paradigm for other phenotypes of post-PE syndrome. Treatment focuses largely on optimizing aspects of the ongoing functional impairment. Pulmonary rehabilitation has been shown to improve functional status in non-PE cohorts including following acute exacerbation of COPD and acute myocardial infarction. The utility of high-intensity interval training in an intermediate–high-risk post-PE population was investigated in a 24-patient randomized clinical trial. The group that received 8 weeks of high-intensity interval training recorded significant improvements in estimated maximal oxygen uptake, forced expiratory volume (FEV1), RV/LV ratio diameter, and health-related quality of life using the SF-36 survey [54]. Similarly, a 3-month exercise and weight loss program demonstrated a significant improvement in peak exercise capacity in the intervention group compared to the control group in patients with recent acute VTE. Symptomatic patients following acute PE diagnosis may also benefit from home-based physical therapy. One hundred and forty patients who received home-based physiotherapist-guided therapy showed improvements in the shuttle walk test and quality of life surveys as measured by Pemb-QoL and the EuroQol-5 questionnaire [55].

Patients with persistent psychological symptoms following PE may benefit from psychotherapy. Cognitive behavioral therapy (CBT) has been shown to reduce intrusive thoughts, improve coping mechanisms, and restore a sense of safety. Supportive psychotherapy and structured patient education regarding recurrence risk, anticoagulation therapy, and prognosis can further reduce anxiety and improve self-efficacy. Integration of mental health specialists within multidisciplinary pulmonary embolism response teams (PERTs) and dedicated post-PE clinics may optimize patient outcomes. Future research should prioritize elucidating mechanisms, refining screening tools, and evaluating targeted interventions to mitigate long-term psychological morbidity in this vulnerable population.

Post-PE patients have a higher risk of coronary artery disease (CAD) compared to age-matched cohorts [56]. Thus, screening for CAD, especially in symptomatic post-PE patients, should be considered. Successful management via revascularization is likely to improve symptoms and functional status. Proper medical management of existing heart failure regimens and arrhythmias may have been altered during acute inpatient admissions for PE and should be reevaluated as indicated [57].

Despite being the preferred treatment for CTEPH, pulmonary thromboendarterectomy (PTE) has been studied less in the CTEPD population. In 42 patients with image-proven CTEPD with mPAP < 25 mmHg that underwent PTE, there was symptomatic improvement in 95% of patients in NYHA I or II groups at 6 months post procedure. Additionally, there was improvement in the median CAMPHOR score with respect to symptoms, activity, and QOL measurements [58]. These clinical improvements were comparable to CTEPH patients undergoing PTE as seen in the PEACOG trial [59]. PTE was noted to be safe in this group, with a 95% 1-year survival rate; however, risks and benefits should be weighed given the estimated 5-year survival of patients with CTEPD of 95% [58,60].

Balloon pulmonary angioplasty (BPA) has been an increased treatment option for patients with inoperable CTEPH. The use of BPA in CTEPD patients has been studied in a small cohort of 10 patients with image-proven CTEPD and mPAP < 25 mmHg. In this cohort, the 6MWT improved by an average of 31m (8.5% improvement from baseline) and patients experienced a decrease in PVR. RV function measured by cardiac MRI was unchanged [61]. Similarly, in another small cohort of 15 CTEPD patients, BPA significantly improved hemodynamics and the 6MWT and lowered the prescribed home oxygen therapy [62]. Further studies are needed to assess the utility of BPA in inoperable CTEPD. Currently, there are no published studies on the use of pulmonary vasodilators in PPES.

## 6. Conclusions and Future Directions

At the heart of managing PPES lies the critical task of identifying its defining characteristics: residual clot burden, right ventricular dysfunction, and exercise intolerance. Tackling these aspects necessitates comprehensive, large-scale, long-term cohort studies aimed at uncovering the risk factors for its emergence following the acute phase. Such research is crucial, especially in evaluating how initial acute PE treatments might affect the development of the syndrome. The ongoing clinical trials investigating innovative treatment devices for intermediate- and high-risk acute PE hold promise in this area, particularly the ones with long-term follow-up. Additionally, the availability of diagnostic algorithms provides essential support for clinicians in identifying PPES phenotypes, with an upcoming large-scale observational study expected to validate the SEARCH algorithm, representing a significant leap forward. A push toward standardizing the definitions of PPES will foster a more uniform approach, enhancing patient guidance on symptom management and follow-up.

To conclude, the literature clearly indicates that post-PE impairment is common across a spectrum of manifestations, emphasizing the critical need for physician awareness at all levels. The complex nature of post-PE conditions, coupled with a still-developing understanding of their phenotypes, necessitates an immediate call for expert consensus. This collaborative effort is vital for navigating the current medical landscape and guiding clinical practice effectively, ultimately aiming to confront the challenges of post-PE syndrome and enhance patient care.

## Figures and Tables

**Table 1:** Phenotypes of post-pulmonary embolism syndrome.

Phenotype	Definition
Post-PE Dyspnea or Functional Impairment	Persistence of dyspnea, exercise intolerance, or decreased functional status after an acute PE, in the absence of an alternate explanation
Post-PE Cardiac Impairment	Persistent right ventricular hypokinesis, dysfunction, or dilation without evidence of pulmonary hypertension after an acute PE in the absence of an alternate explanation
Chronic Thromboembolic Disease (CTEPD)	Persistent symptoms of dyspnea, exercise intolerance, or decreased functional status with evidence of persistent thrombosis without pulmonary hypertension at rest but possible evidence of pulmonary hypertension with exercise
Chronic Thromboembolic Pulmonary Hypertension (CTEPH)	Persistent thrombosis with resting pulmonary arterial hypertension at rest (mean pulmonary artery pressure (mPAP) above 20 mmHg, pulmonary artery wedge pressure (PCWP) of less than 15 mmHg, and pulmonary vascular resistance (PVR) equal or greater than 2 wood units (WU) on a right heart catheterization (RHC))

**Table 2:** CPET abnormalities in post-PE syndrome.

VO_2_ Peak < 80% Predicted
Mild/Moderate Ventilatory Inefficiency: V′_E_/V′_CO2_ Slope or Nadir ≥ 30 and <36
Severe Ventilatory Inefficiency: V′_E_/V′_CO2_ Slope or Nadir ≥ 36
Mild/Moderate Cardiocirculatory Insufficiency: Peak O_2_ Pulse ≥ 70% and <80% Predicted with RER > 1.05
Severe Cardiocirculatory Insufficiency: Peak O_2_ Pulse < 70% Predicted with RER > 1.05

**Table 3:** Findings of diagnostic tests in post-PE syndrome phenotypes.

Diagnosis	Symptoms	CPET	VQ Scan	Echo	CTA	RHC
Post-PE Dyspnea or Functional Impairment	+	Normal or Abnormal	Normal	Normal	Normal	Normal
Post-PE Cardiac Impairment	+/−	Normal or Abnormal	Normal	Abnormal	Normal	Normal
CTEPD	+/−	Normal or Abnormal	Abnormal	Normal or Abnormal	Abnormal	Normal
CTEPH	+	Abnormal	Abnormal	Abnormal	Abnormal	Abnormal

## Data Availability

No new data were created or analyzed in this study. Data sharing is not applicable to this article.

## Abbreviations

6MWD	Six-Minute Walk Distance
BMI	Body Mass Index
BPA	Balloon Pulmonary Angioplasty
CTA	Computed Tomography Angiography
CTEPD	Chronic Thromboembolic Pulmonary Disease
CTEPH	Chronic Thromboembolic Pulmonary Hypertension
CO	Cardiac Output
ESC	European Society of Cardiology
FEV1	Forced Expiratory Volume in One Second
LV	Left Ventricle
mMRC	Modified Medical Research Council
mPAP	Mean Pulmonary Artery Pressure
NYHA	New York Heart Association
PCWP	Pulmonary Capillary Wedge Pressure
PE	Pulmonary Embolism
PEA	Pulmonary Endarterectomy
PEmb-QoL	Pulmonary Embolism Quality of Life
PH	Pulmonary Hypertension
PPES	Post-Pulmonary Embolism Syndrome
PVR	Pulmonary Vascular Resistance
QoL	Quality of Life
RA	Right Atrium
RHC	Right Heart Catheterization
RV	Right Ventricle
RVPO	Residual Pulmonary Vascular Obstruction
SF-36	Short Form 36
SPECT	Single-Positron Emission Computed Tomography
TTE	Transthoracic Echocardiogram
TRV	Tricuspid Regurgitation Velocity
V/Q	Ventilation/Perfusion Scan
VD/VT	Dead-Space Fraction
VE/VCO_2_	Ventilation as a Function of Exhaled CO_2_
VO_2_	Rate of Oxygen Use
